# Environmental Pollution from Illegal Waste Disposal and Health Effects: A Review on the “Triangle of Death”

**DOI:** 10.3390/ijerph120201216

**Published:** 2015-01-22

**Authors:** Maria Triassi, Rossella Alfano, Maddalena Illario, Antonio Nardone, Oreste Caporale, Paolo Montuori

**Affiliations:** 1 Department of Public Health, “Federico II” University, Naples 80131, Italy; E-Mails: triassi@unina.it (M.T.); rossellaalfan@gmail.com (R.A.); antonio.nardone@unina.it (A.N.); oreste.caporale@libero.it (O.C.); 2 Department of Traslational Medical Science, “Federico II” University, Naples 80131, Italy; E-Mail: illario@unina.it

**Keywords:** Campania Region, landfill, incineration, waste, human health, human biomonitoring

## Abstract

The term “triangle of death” was used for the first time by Senior and Mazza in the journal *The Lancet Oncology* referring to the eastern area of the Campania Region (Southern Italy) which has one of the worst records of illegal waste dumping practices. In the past decades, many studies have focused on the potential of illegal waste disposal to cause adverse effects on human health in this area. The great heterogeneity in the findings, and the bias in media communication has generated great healthcare doubts, anxieties and alarm. This paper addresses a review of the up-to-date literature on the “triangle of death”, bringing together the available information on the occurrence and severity of health effects related to illegal waste disposal. The Scopus database was searched using the search terms “waste”, “Campania”, “Naples”, “triangle of death” and “human biomonitoring”. Despite the methodological and sampling heterogeneity between the studies, this review examines the evidence from published data concerning cancer incidence, childhood mortality and birth defects, so that the current situation, knowledge gaps and research priorities can be established. The review aims to provide a contribution to the scientific community, and to respond to the concerns of the general population.

## 1. Introduction

The term “triangle of death” was used for the first time by Senior and Mazza in the journal *The Lancet Oncology* referring to the eastern area of the Campania Region (Southern Italy) which has one of the worst records of illegal waste dumping practices [1]. Since 1980, waste management in Campania region has been characterized by crisis [2]. This waste crisis in the Campania Region has resulted in the widely documented illegal disposal of urban, toxic and industrial wastes [2,3,4,5]. The environmental impacts of illegal waste disposal led to the deterioration of land, as well as ground and surface water, also impacting air quality. 

Waste impact depends on waste composition and illegal disposal practices [6]. Waste composition consists of several types of substances, particularly toxic waste coming from the last phase of the industrial activities: copper, arsenic, mercury, polychlorinated biphenyls, hydrocarbons, *etc.* [4]. Waste disposal practices include illegal burying in areas not legally designated as toxic waste dump sites such as cultivable areas, roads and buildings and construction yards [7]. Furthermore, in the “triangle of death” area, illegal waste burning and the fires set up by residents to burn garbage bags piled up in the streets have contributed significantly to the increase in environmental pollution, particularly of dioxins [8]. Environmental pollution of waste dumping affects health through both short and long-term effects [9,10]. Examples of short-term effects are congenital anomalies, asthma and respiratory infection [11,12]. General symptoms such as stress, anxiety, headache, dizziness, nausea, eye and respiratory irritation have been also described [13]. Long-term health effects related to waste exposure include chronic respiratory and cardiovascular diseases, cancer and even brain, nerves, liver, lymphohematopoietic or kidneys diseases [14,15,16].

In the past decades, many studies in the area of “triangle of death” have focused on the potential health effects of illegal waste disposal, but they have generated heterogeneous results. Some authors, such as Senior and Mazza [1], concluded that the high level of cancer mortality in the area can be linked to the level of pollution caused by inadequate waste-control methods and by illegal dumping. Others discount any significant impact of waste mismanagement on public health [17,18,19,20]. This heterogeneity among studies and especially bias in media communication generated healthcare doubts, anxieties and alarms among residents that have organized committees and associations [21]. In recent years, these committees and associations have consistently raised the issue of the health effects resulting from the waste crisis in Campania, and many disputes and disagreements took place among local governance structures and the population. In order to solve this problem, in March 2014 the Italian Government adopted a special decree, which allocated 25€ million yearly (for 2014 and 2015) for “health screening” of Campania residents. Based on a systematic review of the up-to-date “triangle of death” literature, the present study brings together the information on the occurrence, severity and potential health effects of illegal waste disposal., This review analyzes the evidence from published data concerning cancer, childhood mortality, birth defects and human biomonitoring, despite the methodological and sampling heterogeneity between studies, in order to establish the current situation, knowledge gaps and research priorities. Thus, this review aims not only to provide a contribution to the scientific community, but also to contribute to address the concerns of the population.

## 2. Materials and Methods

The review was conducted in two stages. In the first stage, articles were retrieved via online Scopus database searching. The following keywords and combinations of keywords were used: “waste”, “Campania”, “Naples”, “triangle of death” and “human biomonitoring”. Articles were limited to those that were published in English/Italian-language journals from January 2001 to August 2014. During the second stage, titles and abstracts of articles were independently reviewed by two researchers to assess eligibility for inclusion. If there was any uncertainty, the full text article was retrieved. Disagreements were solved after discussion with a third researcher. Primary publications on waste-related health effects and human biomonitoring studies in the population living in Campania Region were the subject of this review. Other types of papers (environmental impact estimate, waste management, environmental sentinels) were only consulted in order to integrate the relevant available scientific information.

Our initial searches generated 100 hits, which were screened leading to removal of 87 records, leaving 13 full text articles that were evaluated. In addition, four more articles, traced through references listed in review articles, were included. For each of the considered health outcome, the results obtained on Campania residents were compared to the health effects in residents living near landfills and incinerators [9,10,13,22]. Even though in Campania no incinerator has been operating since 2009, we referred to the literature on incinerators for the extensive practice of illegally setting fires to urban and hazardous waste.

## 3. Results 

**Figure 1:**
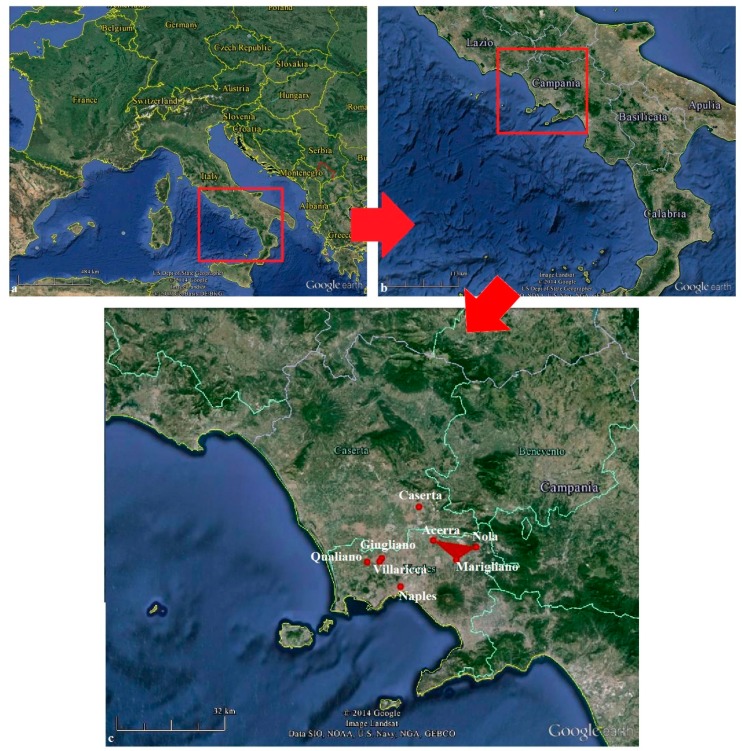
Maps of the study area. (**a**) Italy; (**b**) Campania Region (southern Italy); (**c**) Location of main municipalities under study in southern part of Caserta Province and in the northern part of Naples Province, including the “triangle of death” marked with red.

Seventeen papers (only one review) on health effects of waste exposure and human biomonitoring in Campania were evaluated. Areas under study included: Naples province; Caserta province; Giugliano, Qualiano; Villaricca and the “triangle of death” (between Acerra, Nola and Marigliano) (Figure 1). Ten papers investigated health effects. All cancers, specific cancers, childhood mortality and birth defects were the health outcomes we considered. All cancers, neoplasms of liver, lung, larynx, bladder, leukemia and lymphoma were evaluated by seven studies; colorectal cancer, sarcoma, childhood mortally and birth defects were evaluated by six studies; gastric and kidney cancer were evaluated by five studies. In some papers multiple outcomes were evaluated. Seven papers focused on human biomonitoring: five investigated biomarkers of exposure and two investigated biomarkers of early effect. The findings of these studies are mainly consistent with the previous review by Barba *et al.* [23]. Two summary tables for health effects (Table 1) and human biomonitoring (Table 2), respectively, are presented to show the results.

### 3.1. Studies on Health Outcomes

#### 3.1.1. All Cancers

The mortality observed in the other two municipalities was higher than expected by regional rates, especially for females, even if it did not reach statistical significance [3]. Another ecological study reported an excess of cancer death in the provinces of Naples and Caserta (196 municipalities) compared with the expected deaths from regional rates, also confirmed by Bayesan estimators (Naples: SMR = 106.1 for males; SMR = 107.3 for females; lower limit of 95% CI > 100. Caserta: SMR = 102.5 for males; SMR = 102.9 for females; lower limit of 95% CI > 100) [17]. Furthermore, the cancer mortality rate showed by Senior and Mazza in men living in the so called “triangle of death” was higher than the regional standardized death rate (SDR) 321.7 *vs*. 301.8 per 100,000), even if no confidence interval or standard error were reported [1]. None of these studies evaluated the exposure assessment of the populations under study, the distance from legal or illegal waste sites or other confounders. Information about exposure has been provided only by a trend analysis conducted in Caserta and Naples municipalities [24]. Waste exposure was assessed through the waste exposure index (WEI), an index of environmental pressure due to waste dumping activities described by Musmeci *et al.* [25]. The analysis revealed a statistically significant excess of risk for all cancer of 6.6% (95% CI = 0.8–12.7) only for women living in the municipalities at highest WEI compared with baseline [24]. SENTIERI project revealed a significant mortality risk for all cancers in the contaminated site of National concern composed by 77 municipalities of Caserta and Naples Provinces when compared to mortality expected from the Italian rate, also after adjusting for deprivation index (DI) [SMR DI = 109 (90% CI = 108–111) in males; SMR DI = 105 (90% CI = 102–107) in females] [26]. On the contrary, no statistically significant increase of mortality risk for total cancers was found by two other studies [19,27], considering both WEI and DI.

These results are in contrast with recent evidences on health effect associated with the management of solid waste. Indeed, for total cancers two reviews support an inadequate level of evidence to indicate a role for solid waste [9,10,13], whereas limited [9] or inadequate [13] evidence has been reported for incinerators. 

#### 3.1.2. Liver 

A geographical study pointed out a very high statistically significant increase of mortality for liver cancer in females (SMR = 181.13; lower limit of 95% CI > 100) in a municipality characterized by multiple dumping sites, compared to Campania Region rates [3]. Senior and Mazza described very high mortality rate for liver cancer in the “triangle of death” compared with rates seen in Campania (SDR 35.9 *vs.* 15 per 100,000 in men; 20.5 *vs*. 8.5 in women), but no confidence interval was reported [1]. Comba *et al.* reported excesses of death for liver cancer in Naples province compared with the expected deaths from regional rates (SMR = 117.6 in men; SMR = 114.1 in women; lower limit of 95% CI > 100), while statistical significance was not reached for Caserta Province [17]. A further cluster analysis of the same municipalities revealed three clusters of liver cancer in the southern part of Caserta Province and in the northern part of Naples Province [19]. The analysis was standardized by a deprivation index taking into account education, unemployment, housing ownership, surface of dwelling and family structure as possible socioeconomic confounders. In addition, a trend analysis confirmed an excess risk for liver cancer of 19.3% (95% CI = 1.4–40.3) and 29.1% (95% CI = 7.6–54.8) in men and women respectively in municipalities at highest environmental pressure assessed by waste index category compared with baseline [24]. A significant increase of risk of liver cancer, in term of standardized incidence ratios (SIR), hierarchical Bayesian estimators (BIR) and cluster analysis (BIR = 1.41, 95% CI = 1.17–1.68; BIR = 1.57; 95% CI = 1.28–1.89), was also detected in a two municipalities located in the northern part of Naples Province [27]. The study also detected a positive trend across WEI groups, but statistical significance was not reached. SENTIERI project revealed a significant mortality risk for liver cancer in the contaminated site of National concern composed by 77 municipalities of Caserta and Naples Provinces [SMR adjusted for DI = 125 (90% CI = 117–134) in males; SMR adjusted for deprivation =126 (90% CI = 114–140) in females] [26]. Remarkably, none of the reviewed studies took into account confounders (alcohol, smoking, hepatitis) other than socioeconomic ones, which strongly influence liver cancer development in the province of Naples in particular [28], where HCV and HBV infections are widespread [29].

The association between liver cancer, waste disposal and incinerator has been recently disconfirmed by several evidences reported in the literature [9,30], although a review found limited association with proximity to incinerator plants [10].

#### 3.1.3. Lung 

Altavista *et al.* pointed out a statistically significant increase of mortality for lung cancer especially for females in two out of three municipalities under study compared to Campania Region rates (SMR:1st municipality = 121.85, 2nd municipality = 120.94 for males; SMR 2nd municipality = 176.94 for females; lower limit of 95% CI > 100) [3]. Similar results were reported by four other studies: Comba *et al.* revealed excess of death for lung cancer in the provinces of Naples and Caserta compared with the expected deaths from regional rates [SMR = 114.1 (males)–126.5 (females); lower limit of 95% CI > 100] [17]; two other studies showed two clusters of lung cancer in the southern part of Caserta Province and in the northern part of Naples Province [19,27]; a trend analysis revealed only in males of the same municipalities of Naples and Caserta a statistically significant excess relative risk of (95% CI = 0.4–3.3) of death for lung cancer with the increase of waste index category [24]. In contrast, Senior and Mazza described a mortality rate for lung cancer in the “triangle of death” equivalent to the rates seen in Campania (SDR 97.8 *vs*. 97.4 per 100,000 in men; no CI or standard error reported) [1] and no excess of death for lung cancer arose from SENTIERI project [26]. All the studies didn’t evaluate smoking habits or other confounders and are therefore difficult to interpret. No association between lung cancer, waste disposal and incinerators has nowadays been described in the literature [9], even if a review found a limited association with proximity to incinerator plants [10]. 

#### 3.1.4. Larynx 

Altavista *et al.* pointed out an increase of mortality for larynx cancer in three municipalities characterized by multiple dumping sites, compared to Campania Region rates, but statistical significance was reached only in one municipality for males (SMR = 211.85; lower limit of 95% CI > 100) and in one municipality for females (SMR = 339.42; lower limit of 95% CI > 100) [3]. Senior and Mazza described a higher mortality rate for larynx cancer in men living in the “triangle of death” compared with Campania rates (SDR 12.8 *vs*. 8.7 per 100,000; no CI or ES reported) [1]. Another ecological study confirmed the increase of mortality for larynx cancer in males only in the province of Naples (SMR = 111.8; lower limit of 95% CI > 100) [17]. Furthermore, the SENTIERI project revealed a statistically significant mortality risk for larynx cancer in males living in the contaminated site of national concern composed by 77 municipalities of Caserta and Naples Provinces [SMR adjusted for deprivation = 115 (90% CI = 105‒127)] [26]. Even if data are concordant, no study designs evaluated waste pressure in the residence areas and only the SENTIERI study took into account socio-economic deprivation. Furthermore, these results are in contrast with the evidence summarized by two reviews [9,10] and with the evaluation by the epidemiological evidence of SENTIERI project that showed no association between larynx cancer and landfills or incinerators [22]. 

#### 3.1.5. Bladder 

Studies on bladder cancer are discordant. A geographical study pointed out a significant increase of mortality for bladder in males living in a municipality characterized by multiple dumping sites, compared to Campania Region rates (SMR = 130.12; lower limit of 95% CI > 100) [3]. Senior and Mazza described a higher mortality rate for bladder cancer in men living in the “triangle of death” compared with rates seen in Campania, while bladder cancer risk in women appeared lower than regional rates (SDR 29.3 *vs*. 21.7 per 100,000 in males; 3.1 *vs*. 4,2 per 100,000 in females), even if no confidence interval was reported [1]. Comba *et al.* reported excess of death for bladder cancer in the provinces of Naples and Caserta compared with the expected deaths from regional rates (SMR = 110.7 in males; 117.5 in females; lower limit of 95% CI > 100) [17]. A cluster analysis of the same municipalities revealed two significant clusters of bladder cancer in the southern part of Caserta Province and in the northern part of Naples Province [19]. Three other studies revealed no risk of death for bladder cancer in the same areas, adding information about intensity of waste-related exposure and socio-economic deprivation [24,26,27].

Although not conclusive, these data are consistent with the literature on bladder cancer: two reviews [9,10], the evaluation of the epidemiological evidence of SENTIERI project [22] and one study on 15 landfills highlight an inadequate level of evidence to indicate a role for solid waste and incinerators [30].

#### 3.1.6. Stomach

A statistically significant decrease in mortality for gastric cancer was found by a geographical study in males of a municipality of Naples characterized by multiple dumping sites compared to Campania Region rates (SMR = 56.1; upper limit of 95% CI < 100) [3]. No risk arose from SENTIERI project in the contaminated site of National concern composed by Caserta and Naples province [26]. By contrast, Comba *et al.* reported statistically significant excess of death for gastric cancer in males living in the province of Caserta compared with the expected deaths from regional rates (SMR= 129.3 in men; SMR = 118.2 in women; lower limit of 95% CI > 100) [17]. A cluster analysis of the same municipalities detected a big cluster of gastric cancer in the north-western part of Naples and south-western part of Caserta Province, mainly due to male mortality [19]. These results were confirmed by a trend analysis which revealed in men living in 35 municipalities of Caserta and Naples a statistically significant excess relative risk (5.2%; 95% CI = 1.8‒8.7) of death for gastric cancer with the increase of waste index category [24].

No association between gastric cancer and waste disposal and incinerator is supported by the last evidences in the literature [9], even if two less recent studies found limited association with proximity to incinerator plants [10,22].

#### 3.1.7. Colorectal

The only study suggesting an increase of risk of colorectal cancer in the Campania Region is the report of Senior and Mazza that described a higher mortality rate for colorectal cancer in women in the “triangle of death” compared with rates seen in Campania (SDR 29 *vs.* 26.4 per 100,000), but no confidence interval was reported [1]. No association was found by five other different studies [3,17,19,26,27]. These results are consistent with the evidence summarized by a review, that argues the association between colorectal cancer and waste disposal and incinerators [9], although a less recent review found limited association with proximity to incinerator plants [10].

#### 3.1.8. Leukemia and Lymphoma

Data reported by Senior and Mazza described higher mortality rates for leukemia in males living in the “triangle of death” compared with rates seen in Campania (SDR 13.1 *vs*. 10.1 per 100,000), and higher leukemia and lymphoma in both sexes living in this area than in the rest of Campania area, and were referred to the “Local Health District Naples 4” [SDR 28.2 *vs*. 17.9 (in males); 18.7 *vs.* 16.1 (in females) per 100,000], even though the study didn’t provide any information about confidence interval or exposure data [1]. On the other hand, no statistically significant excess of risk for leukemia and lymphoma was found by Altavista *et al.* [3], nor was it found for non-Hodgkin lymphoma (NHL) by Fazzo *et al.* [19]. Similar results were provided by another geographical study, except for an excess of risk (SMR = 109.1; lower limit of 95% CI > 100) of NHL, found only in women living in the province of Naples [17]. Taking into account the deprivation index, a study found statistically significant excess of relative risk (5.7%; 95% CI = 0.2‒11.5) of death for NHL only in men with the increase of waste index category [24]. Another study identified a statistically significant cluster of leukemia in the total population of Naples Province [relative risk (RR) = 1.33; *p*-value = 0.05], as well as a significant increase of risk for leukemia and NHL (in terms of SIR, however it was not confirmed by BIR analysis) [27]. The SENTIERI Project found no significant increase of risk for leukemia or lymphoma in the contaminated site of National concern composed by 77 municipalities of Caserta and Naples Provinces [26].

Data from the literature are not conclusive with respect to association of leukemia and NHL with waste management: no association between leukemia, NHL and landfills or incinerators is supported by a recent review [9], while one study detected a significant risk for leukemia in residents near benzene waste sites [31], and three more studies found limited association of NHL with proximity to incinerator plants [10,22,30].

#### 3.1.9. Kidney

An ecological study reported statistically significant excess of death for kidney cancer in females living in the province of Naples compared with the expected deaths from regional rates (SMR = 120.7; lower limit of 95% CI > 100) [17]. A cluster analysis found a cluster of kidney cancer in the total population, located in the north-western part of Naples Province [19]. No excess of risk for kidney cancer was found by three other studies, that also evaluated waste-exposure pressure and socioeconomic confounders [24,26,27]. These results are consistent with recent literature on the health impact of landfills and incinerators, that found inadequate evidence for a link to kidney cancer [9,10].

#### 3.1.10. Soft-Tissue Sarcoma

A cluster analysis on 35 municipalities of Naples Province served by a Cancer Registry, revealed a statistically significant cluster of soft-tissue sarcoma (STS), even if no correlation with waste exposure index was detected [27]. Two previous geographical studies [17,19] and a trend analysis showed no statistically significant increased risk of cancer death for STS [24]. A more recent geographical study focused on the topic and found no significant increase in incidence of STS other than for gastrointestinal stromal tumors (GIST) in males [SIR 2.04 (overall), 95% CI = 1.26‒3.11; SIR 2.20 (males), 95% CI = 1.10‒3.94; not significant SIR 1.88 (females), 95% CI = 0.90‒3.46] [32].

No evidence of association between STS and landfills is nowadays supported by the literature, whereas limited association has been reported for people living in proximity of incinerators, in relation to emissions of dioxins [9,10,22].

#### 3.1.11. Childhood Mortality and Malformations

In the first pilot study on health effects to waste exposure in communities living in the Campania Region, a significant risk increase was found for low birth weight, fetal distress and infantile cancers in residents of some municipalities of Caserta, where waste dumping, both legal and illegal, were mainly located [5]. Similar results arose from three studies: an ecological study reported increases of observed birth defects, especially cardiovascular and urogenital malformations, in the provinces of Naples and Caserta compared to the expected ones based on regional rates [17]. A cluster analysis of the same area revealed statistically significant clusters of total congenital malformations (five clusters), cardiovascular (two clusters), urogenital (three clusters) and limb malformations (only one cluster) [19]. A trend analysis of the same municipalities revealed a statistically significant increasing trend for urogenital anomalies [excess relative risk (ERR) = 13.8; 95%CI = 5l8 to 22l5] and a decreasing trend for cardiovascular anomalies (ERR = −5.3; 95% CI = −9.4 to −1). Comparisons between highest exposure waste municipalities and baseline were statistically significant for urogenital (ERR = 82.7; 95% CI = 25.6–165.7) and nervous system malformations (ERR = 83.5; 95% CI = 24.7–169.9) [24]. By contrast, no risk for congenital malformations was detected by Altavista *et al.* in populations living in three municipalities of Campania Region characterized by multiple dumping sites, although no information about exposure assessment or confounders was provided [3]. The SENTIERI project detected no risk of death for congenital malformations for all ages in the contaminated site of National concern composed by 77 municipalities of Caserta and Naples Provinces and even a statistically decreased in risk when considering only 0–1 year children [SMR adjusted for deprivation = 97 (90% CI = 88–106) for all ages; SMR adjusted for deprivation = 90 (90% CI = 83–97) for 0–1 year children] [26]. 

Recent reviews on the potential health hazards of waste management found a limited evidence of association between congenital malformations and living near landfills (of urogenital and nervous systems) and incinerators (urogenital and orofacial) [9,11,13], although two previous reviews found a limited evidence of higher risk of congenital anomalies in residents near landfills, and inadequate evidence for people living near incinerators [10,22]. 

### 3.2. Studies on Human Biomonitoring

Six studies investigated the link between waste-related pollution and biomarkers assessment in humans. Two studies identified high dioxin levels in breast milk of 94 primiparae living in Naples and Caserta provinces, and detected a positive correlation with age of sampled women, illegal waste fires and environmental dioxin risk index (EDR), that is an index based on dioxins concentrations in buffalo milk samples [33,34]. Illegal waste fires appeared to be a more important determinant of dioxin exposure in milk for women living in an area at low risk compared to high risk areas. Moreover, shorter telomere length and lower telomerase activity were found in peripheral blood mononuclear cells of healthy pregnant women living in north-east Naples area, who were affected by intense waste pressure compared to women living in non-polluted area [35]. In a study on assessment of DNA damage by Random Amplification of Polymorphic DNA (RAPD) in *Paracentrotus lividus* embryos exposed to amniotic fluid, different polymorphisms were found in embryos exposed to amniotic fluid from 15 residents living in north-east Naples area compared with those related non-polluted area citizens (Avellino), giving evidence that pollution levels in the “triangle of death” adversely affected human amniotic fluids [36]. On the contrary, a more recent study found PCDD/Fs and PCBs serum level in the population living in the Naples area that were lower than current values observed in populations living in exposed areas. Moreover the study detected no significant differences between serum dioxin concentrations in people living in “triangle of death” and its surroundings [18]. Similarly, a study on women living in Giugliano, a municipality affected by open-air waste combustion accidents, found PCDD/Fs and PCBs levels in breast milk significantly lower compared with those of donors from two cities highly polluted, that were Piacenza and Milan (Italy) [20]. Furthermore, the SEBIOREC study found biomarkers’ concentrations in Campania region compatible with European and Italian current values, excepted for a relative overexposure to some pollutants (As, Hg, dioxins) detected in four municipalities of Naples characterized by high waste pressure [37]. 

**Table 1:** Summary table of studies on health effects.

Authors	Study Design	Study Subjects (Years of Observation)	Exposure	Confounders	Health Outcames	Reported Findings
Trinca *et al.* (2001) [5]	Geographical study	Infant residents in **104** municipalities of Caserta (1985–1994)	Spatial distribution of sites using GIS	None	Childhood morbidity and mortality	- Low birth weight - Fetal distress - Infantile cancers
Altavista *et al.* (2004) [3]	Geographical study	150000 residents in **3** municipalities of Naples (Giugliano, Qualiano and Villaricca) (1986–2000)	Spatial distribution of sites using GIS	None	Cancer mortality and congenital malformations	- All cancers SMR = 107.23 (M) – 111.08 (F) - Liver SMR = 181.13 (F) - Lung SMR = 121.85 (M) – 176.94 (F) - Larynx SMR = 211.85 (M) - Bladder SMR = 130.12 (M) - Stomach SMR = 56.1 (M)
Senior and Mazza (2004) [1]	Geographical study	250000 residents in **District 73**, known as Triangle of death (2002)	n/a	None	Cancer mortality	- All cancers SDR = 321.7 per 10^5^ (M) *vs.* regional rate 301.8 - Liver SDR = 35.9 per 10^5^ (M) *vs.* regional rate 20.5 - Larynx SDR = 12.8 per 10^5^ (M) *vs.* regional rate 8.7 - Bladder SDR = 29.3 per 10^5^ (M) *vs.* regional rate 21.7 - Colorectal SDR = 29 per 10^5^ (M) *vs.* regional rate 26.4 - Leukemia and lymphoma SDR = 28.2 per 10^5^ *vs.* regional rate 17.9 (M); 18.7 per 10^5^ *vs.* regional rate16.1 (F)
Comba *et al.* (2006) [17]	Geographical study	About 4 million residents in **196** municipalities of Caserta and Naples (1994–2001) ^a^	Distance of residence	None	Cancer mortality and congenital malformation	- All cancers SMR = 106.1(M) – 107.3 (F) - Liver SMR = 117.6(M) – 114.1 (F) - Lung SMR = 114.1 (M) – 126.5 (F) - Larynx SMR = 111.8 (M) - Bladder SMR = 110.7 (M) – 117.5 (F) - Stomach SMR = 129.3 (M) – 118.2 (F) - NHL SMR = 109.1 (F) - Kidney SMR = 120.7 (F) - Malformations: total, cardiovascular and urogenital
Fazzo *et al.* (2008) [19]	Cluster analysis	Residents in **196** municipalities of Caserta and Naples (1994–2001) ^a^	n/a	Deprivation index (DI)	Cancer mortality and congenital malformation	- Liver (nr.4 cluster) RR = 2.04, *p*-value = 0.0003 - Lung (nr. 3 cluster) RR = 1.30, *p*-value = 0.0003 - Bladder(nr. 2 cluster) RR= 1.44, *p*-value = 0.004 - Stomach (nr.1 cluster) RR = 1.31, *p*-value = 0.05 (M) - Kidney (nr.1 cluster) RR = 1.70, *p*-value = 0.01 - Malformations: total, cardiovascular, urogenital and limb
Martuzzi *et al.* (2009) [24]	Geographical study, cluster analysis	About 4.9 million of residents in **196** municipalities of Caserta and Naples (1994–2001) ^a^	Waste index category (WIC)	DI,	Cancer mortality and congenital malformation	- All cancers ERR = 6.6% (F) - Liver ERR = 19.3% (M) – 29.1 (F) - Lung ERR = 1.9% (M) - Stomach ERR = 5.2% (M) - NHL ERR = 5.7% (M) - Malformations, especially urogenital (ERR = 82.7%) and nervous system (ERR = 83.5%)
Fazzo *et al.* (2011) [27]	Cluster analysis	About 5 million residents in **35** municipalities of Naples Province (1997–2005) ^b^	WIC	DI	Cancer mortality and congenital malformation	- Liver RR = 1.64, *p*-value = 0.0003 - Lung RR = 1.15, *p*-value = 0.08 - Leukemia RR = 1.33, *p*-value = 0.05 - Soft-tissue sarcoma RR = 2.02, *p*-value = 0.08
Pirastu *et al.* (2011) [26]	Geographical study	Residents in **77** municipalities of Caserta and Naples (1994–2001) ^a^	Distance of residence	DI	Cancer mortality and congenital malformation	- All cancers SMR DI = 109 (M) – 105(F) - Liver SMR DI = 125 (M) – 126 (F) - Larynx SMR DI = 115 (M)
Benedetti *et al.* (2013) [32]	Geographical study	Resident in **35** municipalities of Naples Province served by a Cancer Registry (1997–2005) ^b^	n/a	None	Soft tissue sarcomas	Gastrointestinal stromal tumors SIR = 2.04 (overall) –2.20 (M)

M = male; F = female; ^a^ studies that draw their data from the same database. ^b^ studies that draw their data from the same database.

**Table 2:** Summary table of studies on human biomonitoring.

Authors	Donors	Samples	Biomarkers Studied	Exposure	Confunders	Reported Findings
Guida *et al.* (2010) [36]	15 woman living in north-east Naples area *vs.* 15 woman living in a non-polluted area (Avellino, Campania)	Individual samples	Embryotoxcity, spermiotoxicity assay and RADP profile of sea urchin embryos DNA after the exposure to polluted/non polluted amniotic fluid	n/a	none	Significant spermiotoxicity and embryotoxcity anddifferent RAPD polymorphism were observed for polluted amniotic liquid-treated sea urchin samples compared to non polluted samples
Ulaszewska *et al.* (2011) [20]	59 healthy mothers (21 mothers from Giugliano, 22 from Piacenza and 16 from Milan)	Individual samples	PCDD-Fs and dl-PCBs in breast milk	n/a	Diet, age, number of deliveries and smoking	PCDD/F, PCB levels and WHO-TEQs in milk samples collected in Giugliano were lower than those taken in Piacenza and Milan
De Felice *et al.* (2012) [35]	50 woman living in north-east Naples area *vs.* 50 controls from non-polluted areas	Individual samples	Telomere length and telomerase activity in mononuclear blood cells	n/a	Age, number of deliveries, weeks of pregnancy	Residing in polluted areas is significant associated with lower telomere length and telomerase activity
Rivezzi *et al.* (2013) [34]	95 women had been living in Caserta and Naples provinces at least for previous 10 years	Individual samples	PCDD-Fs and dl-PCBs in breast milk	Waste index category (WIC), presence of toxic waste or illegal burning of waste near the residence	Age, smoking, cheese consumptio, occupation	High values of dioxins were detected. Positive correlation was found with age and exposure to fires. Exposure to fires was more determinant in low than in high risk area
Giovannini *et al.* (2014) [33]	95 women had been living in Caserta and Naples provinces at least for previous 10 years	Individual samples	PCDD-Fs and dl-PCBs in breast milk	WIC, presence of toxic waste or illegal burning of waste near the residence	Age, smoking, cheese consumptio, occupation	Positive correlation was found with age, EDR (Environmental Risk of Dioxin)and fires
Esposito *et al.* (2014) [18]	22 subjects had been living in “risk area” of Naples for at least 1 year *vs.* 33 control subjects from non polluted areas	Individual samples	Serum PCDD-Fs and dl-PCBs	n/a	Age, body mass index	Biomarkers' concentrations were lower than values detected in studies on populations living in exposed areas
De Felip *et al.* (2014) [37] *Case-control*	For serum and blood samples:859 subjects living in Naples and Caserta; for milk samples: 52 women from same areas	Pooled samples: 84 serum, 84 blood and 6 milk samples	PCDD-Fs,-PCBs, As, Hg, Cd and Pb in blood and serum; PCDD-Fs, PCDEs-PCBs, As, Hg, Cd and Pb in breast milk	n/a	Age range and gender	Biomarkers' concentrations were found to be compatible with their current values in European countries and in Italy

^a^ studies that draw their data from the same database.

## 4. Discussion 

One of the main problems in assessing the impact on health-related waste is the inadequacy of information. In fact, our initial searches generated 100 hits. Most of them deal with waste management, policy, economic evaluation, sociological features or environmental sentinels. The Campania Region has been characterized by a waste crisis since the 80s, hence the presence of such a large number of manuscripts is not surprising, but only 13 of them dealt with health effects and human biomonitoring. Several reasons might account for this: first of all, the best studies to investigate a link between exposure and health effects are descriptive, and use routine data (health statistics in this particular case) from the population supposed to be at risk. Routine data are often available with delay (death statistics 1994–2001 were used to the first time in 2006 [17]), which probably causes a delay in publication of evidences and reduces the probability of finding manuscripts on this specific topic in the literature. In order to define the population at risk, most studies have been focusing on identifying the exact location of waste disposals, that in Campania are often illegal and difficult to find. In addition, biomonitoring studies in human have been performed only recently probably because of difficulty in recruiting donors. Only in 2004 the Civil Protection commissioned a research to a task composed by experts from World Health Organization, from the Italian “Istituto Superiore di Sanità” and from Campania Region to investigate the potential health effect related to waste mismanagement only in 2004.

Although many reviews are available, the lack of data on exposure levels represents the major issue in the evaluation of health effects arising from exposure to waste [9,10,16,38]. Furthermore, in Campania the difficulty in exactly identifying waste arrangement and locating illegal waste dumping, sinking and burning complicates the matter. The population at risk has been hard to identify, and this could represent one of the main causes of delay in publication of evidences about potential health effects related to waste exposure. Residential population have been exposed to a mixture of chemicals, often toxic substances blended in with urban waste, since the 80s, and low exposures, even if extended in time, are usually hard to identify. Exposure was estimated only in two studies through a synthetic index based not only on distance from dumping sites, but also on intrinsic characterization of the waste disposal (toxic and hazardous substances are often blend in with urban waste), on percentage of resident population and on the characterization of neighboring areas by a geographic information system (GIS) [24,27]. Furthermore, the application of this exposure index, performed by Musmeci *et al.* [25], in the study of Fazzo *et al.* [19] finds some limits, as pointed out by the authors, because of the modest consistency between the two correlation studies, that result in lower statistical power. Another limit lies in the fact that the index does not take into account the burden of illegal waste fires as suggested by the findings of Rivezzi *et al.* [34]. Indeed, exposure to fires resulted in larger increases of dioxin concentrations in people living in low risk areas than those from high risk areas [34]. In addition, low-dose exposures affect the relative risk in small increments that are difficult to distinguish from those introduced by confounding factor [16]. No study reviewed or evaluated confounding factors other than socioeconomic ones (education, unemployment, housing ownership, surface of dwelling and family structure), so that any conclusion has to be viewed in the light of variability and uncertainty in the results. Another constraint of the studies on residents in Campania Region is that health data are far from being exhaustive. All the studies presented draw their data from the Cancer Registry of Local Health Authority “Naples 4”, and from the National Hospital Discharge Records database, the National Bureau of Statistics (ISTAT) that are compromised by problems related to codification quality [39], delayed availability and lack of information.

Most of the studies taken into account show only small increases of health risk (RR between 1 and 2) for the residents in Campania. These results are hard to explain also because different effect measures (SMR, RR, ERR, SIR, BIR, SDR) have been used in the studies, so that no direct comparison can be done, even for the same measure (e.g. SMR), and a single result is impossible to summarize. In addition, all the studies investigate multiple effects, which increases the chance of a false positive result (error type 1). Despite these constraints, this review provides new relevant information. Most studies analyzed in the review find a significant increase of *total*, *liver* and *lung* cancer mortality in the Region; even if the study design, the heterogeneity of distribution, the lack of information on confounders different from socioeconomic ones, which are important in such multifactorial pathologies, can support only a possible role rather than a causal inference. All studies on *larynx* show an excess of risk only in men, furthermore no study evaluated confounding factors neither intensity of waste exposure. Some studies reveal an excess of risk for *bladder* and *stomach cancer* only in men, additionally the excess of risk for bladder cancer disappears when considering confounding factors, and no trend risk is revealed comparing areas of different waste-exposure pressure. Similar results have been detected for *kidney cancer* in women. Even if some studies suggest a higher risk of death for this cancer in Campania Region residents, this hypothesis has not been confirmed by more recent studies that evaluated waste-exposure pressure and socioeconomic confounders. Data reported for *leukemia* and *lymphoma* are discordant and non-comparable in both sexes, thus suggesting more a role of other risk factors in the two genders rather than an association with waste exposure. No risk has been detected for *colorectal cancer* and *soft-tissue sarcoma*. The link between higher incidence of *GIST* and living in Campania needs to be clarified, since no given etiological hypothesis still exists, and the mentioned environmental contamination by PCDD/F and PCB has been recently debated [18]. *Congenital malformation* (particularly urogenital) outbreaks in the Region linked to environmental pollution need further investigation, since the evidence up to date only suggests a possible role of waste-related exposure. 

Regarding the biomonitoring, despite several surveys detected bioaccumulation of different pollutants in animals of Campania Region [40,41,42,43,44], evidence on humans are not conclusive. Studies on biomarkers of early effect reveal a possible correlation between pollution levels in the “triangle of death” and adverse effects to human health. The SEBIOREC study limits to dioxins and heavy metals the possible overexposure due to waste mismanagement in the Region, and defines municipalities that might deserve closer health surveillance [37]. Some data reported for dioxin in breast milk indicate that the source of contamination could be the illegal waste burning, since no incinerator was operating when the analysis was conducted [33]. Moreover, a long lasting human exposure could be hypothesized because of correlation with age. By contrast, two more recent studies, even based on a limited number of subjects, show values of serum/milk dioxin lower than other European countries and Italy, thus suggesting that waste related pollution does not have an impact on health as significant as supposed in the last years [18,20]. 

The findings presented so far suggest a possible role for environmental waste in the increasing cancer rates detected in the Region, but evidence for a causal relationship is still weak, mainly due the lack of exposure information and confounders control. Since possible health dangers attracted considerable media attention and protest actions, at the beginning of 2014 Italian authorities issued emergency measures to face growing pressure and guarantee the safety of Campania region. Emergency measures included a dedicated decree (law n.6 6 February 2014), known as “Land of Fires” Decree, that was shaped to tackle environmental and industrial emergencies and to support the development of the most deprived areas. Main targets of the decree were mapping and remediation of contaminated sites, health screening of resident population, territory control and economic recovery. Nevertheless its constraints, the Land of Fires Decree was innovative and proved the real interest of institutions towards citizens. First, the mapping of contaminated land represented not only a necessary action of environmental restoration but also a needful answer to the agri-food industry, that experienced hard times for the simultaneous existence of waste and economic crisis. In fact, media scaremongering generated deep skepticism in both consumers and major brands concerning Campania products, to the point that, to safeguard consumers’ concerns, suppliers begun to provide their raw materials outside of the Region. The decree allowed to classify soils, depending on vulnerability, in “food” and “no food”, pointing out definitively which lands were banned from food production. However, the decree did not find unidirectional answers to Campania’s problems because of critical issues imposed by the crisis itself. For example, the decree introduced the crime of burning waste deposited in areas not intended for landfill, neglecting the issue of special and hazardous waste. The legislature, in fact, would have to be more radical and hard about waste origin, in particular industrial, and factors that cause illegal waste burying or burning as final destination. Finally, other critical issues of particular importance concerned health safety. The decree appropriated 25 billion € of funds yearly (for 2014 and 2015) for “health screening” of residents in Campania and Apulia region, that were recognized by the WHO as high environmental risk areas since 1986 because of their metallurgic industries. The decree hasn’t clarified the meaning of “health screening”. Currently, screenings that have scientific evidence of effectiveness and efficiency are only three: mammography for breast cancer, Pap tests for the cervical cancer and fecal occult blood test for colorectal cancer [45,46,47]. But they may be hasty and inappropriate to be carried out for all Campania residents. We hypothesize that the decree probably refers to biomonitoring activities, based on markers of susceptibility, exposure and early effects, as a first approach to study citizens of affected areas. In fact, even if exposure data on animals are available, biomonitoring studies on humans are only a few. Future studies are warranted to better clarify total burden of exposure to low levels of pollutants in the long range, and to identify early biological effect. Biomarkers are just one avenue to improve the measurement of exposure, susceptibility and disease; the field of cancer epidemiology finds its new challenge in exposomes, that promise to provide further insights in environmental health [48,49] and offer the opportunity to throw light also on Campania region scenario. Further studies are needed to confirm the early effects detected in response to the exposure to waste burning and dumping by De Felice *et al.* [35] and Guida *et al.* [36]. Finally, the “Land of Fires” Decree, in order to deploy its full potential, should represent only the beginning of multiple coordinated and targeted government actions to follow.

## 5. Conclusions 

Although many intrinsic limits affect contrasting studies and data [14,20], overall available findings point out a possible long term role of waste, as suggested by positive correlation with outcomes as liver and lung cancer mortality, in addition to a short term effect waste-related (less than 1 year), confirmed by association with congenital malformation, which is compatible with the lack of remediation of the polluted sites and persistence of waste mismanagement to date. Research on exposure to pollutants confirms a possible exposure to illegal waste fires in the “triangle of death”, nevertheless no risk excess for diseases has been recognized to be related to incineration (sarcomas, non-Hodgkin lymphomas), and has been detected in resident population. Further studies are needed to better define waste related-health effects, since updated data are still far from being conclusive. In spite of methodological and sampling heterogeneity among studies, this review compiles the data in a harmonized and effective way, so that the current status, knowledge gaps and research priorities can be established. Thus, the present review wishes not only to provide a contribution to the scientific community, but also to support recommendations for mitigating pollution sources and risks in the area of concern. A similar process of analysis may be carried out for other areas and pressures in order to facilitate policy making at regional, national as well as at European level. 
